# Copy number alterations and allelic ratio in relation to recurrence of rectal cancer

**DOI:** 10.1186/s12864-015-1550-0

**Published:** 2015-06-06

**Authors:** Inès J Goossens-Beumer, Jan Oosting, Wim E Corver, Marjolein JFW Janssen, Bart Janssen, Wilbert van Workum, Eliane CM Zeestraten, Cornelis JH van de Velde, Hans Morreau, Peter JK Kuppen, Tom van Wezel

**Affiliations:** Department of Surgery, Leiden University Medical Center, Leiden, The Netherlands; Department of Pathology, L1-Q, Leiden University Medical Center, PO Box 9600, 2300 RC Leiden, The Netherlands; ServiceXS, Plesmanlaan 1d, Leiden, The Netherlands

## Abstract

**Background:**

In rectal cancer, total mesorectal excision surgery combined with preoperative (chemo)radiotherapy reduces local recurrence rates but does not improve overall patient survival, a result that may be due to the harmful side effects and/or co-morbidity of preoperative treatment. New biomarkers are needed to facilitate identification of rectal cancer patients at high risk for local recurrent disease. This would allow for preoperative (chemo)radiotherapy to be restricted to high-risk patients, thereby reducing overtreatment and allowing personalized treatment protocols. We analyzed genome-wide DNA copy number (CN) and allelic alterations in 112 tumors from preoperatively untreated rectal cancer patients. Sixty-six patients with local and/or distant recurrent disease were compared to matched controls without recurrence. Results were validated in a second cohort of tumors from 95 matched rectal cancer patients. Additionally, we performed a meta-analysis that included 42 studies reporting on CN alterations in colorectal cancer and compared results to our own data.

**Results:**

The genomic profiles in our study were comparable to other rectal cancer studies. Results of the meta-analysis supported the hypothesis that colon cancer and rectal cancer may be distinct disease entities. In our discovery patient study cohort, allelic retention of chromosome 7 was significantly associated with local recurrent disease. Data from the validation cohort were supportive, albeit not statistically significant, of this finding.

**Conclusions:**

We showed that retention of heterozygosity on chromosome 7 may be associated with local recurrence in rectal cancer. Further research is warranted to elucidate the mechanisms and effect of retention of chromosome 7 on the development of local recurrent disease in rectal cancer.

**Electronic supplementary material:**

The online version of this article (doi:10.1186/s12864-015-1550-0) contains supplementary material, which is available to authorized users.

## Background

The Dutch Total Mesorectal Excision (TME) trial [1] changed standard treatment guidelines for rectal cancer patients [2]. This international trial was designed to provide a clinical assessment of whether additional preoperative short-term radiotherapy (pRT) could reduce the number of local recurrences compared to TME surgery alone. Based on the Dutch TME trial, patients with tumor stage 2 (T2) were recommended to receive pRT in addition to TME surgery [2]. The beneficial effect of TME surgery combined with pRT on rectal cancer local recurrence rates was subsequently confirmed in a Dutch population-based study [3]. In addition to pRT, preoperative concurrent chemoradiation treatment (pCCRT) has been studied internationally and proposed as a preoperative treatment for the reduction of local recurrent disease related to rectal cancer. Reductions in local recurrence achieved by pCCRT were similar to those for pRT [4], and the postoperative complications associated with pRT and pCCRT were also reported to be comparable [5]. In 2013, an EURECCA consensus was published which provided treatment recommendations for rectal cancer aimed at minimizing the differences in rectal cancer treatment regimes in Europe [6,7]. Although both pRT and pCCRT are recommended for specific TNM stages and introduction of these approaches has reduced local recurrence rates markedly, no difference in overall survival has yet been achieved [2,4,8]. This lack of survival benefit is most likely caused by the harmful side effects [9] and co-morbidity of the preoperative treatment [2]. Recently, a phase III clinical trial named RAPIDO was initiated to assess the survival benefit of pRT followed by full-dose preoperative chemotherapy as an alternative for pCCRT with and without postoperative chemotherapy [10].

Under current guidelines, preoperative treatment is offered to a broad group of patients, although only around 10% of patients are actually at risk for development of a local recurrence without preoperative treatment [2]. This means that up to 90% of rectal cancer patients are unnecessarily exposed to preoperative treatment [2]. This undesirable situation calls for effective biomarkers that allow for selection of those patients with a high probability for development of a local recurrence after surgery alone. Effective selection for pRT or pCCRT treatment of only those rectal cancer patients with an increased risk of recurrent local disease would reduce overtreatment and allow for more personalized treatment protocols.

Genetic profiling of copy number (CN) alterations in individual chromosomes has previously been recognized as an independent predictor for metastatic relapse of early stage colorectal cancer [11]. The risk of development of a local recurrence in rectal cancer can be deduced from the genetic aberrations found in the primary tumor. In the current study, we were especially interested in the prognostic value of genomic alterations related to local recurrent disease in rectal cancer. The aim of our study was therefore to identify CN alterations and allelic imbalances that could predict risk of recurrence in rectal cancer. Using a genome-wide CN analysis approach, we analyzed tissues from 112 rectal cancer patients enrolled in the TME trial. High-density SNP arrays were used to assess CN alterations and allelic (im)balances of genomic DNA (gDNA) segments [12,13]. As many studies have reported on CN alterations in (colo)rectal cancer, we additionally performed a meta-analysis of 42 studies that reported chromosomal CN alterations (high frequency CN alterations at the level of chromosome arms) and compared these to our current findings. We hypothesized that the CN alterations and/or aberrant allelic ratio patterns of certain gDNA segments might be prognostic for a local recurrence in rectal cancer, and might therefore identify those patients that would benefit most from pRT or pCCRT.

### Study cohort

Tumor and matching normal tissues from the Dutch TME trial, which recruited rectal cancer patients in 118 European centers and one Canadian center, were available for analysis. The trial design has been described previously [1]. We selected patients (from the non-radiation treatment arm) with clinically resected TNM stage I-III adenocarcinomas of the rectum and a presentation of local and/or distant recurrent disease at follow-up. To exclude uncontrolled bias, patients with clinically resected TNM stage I-III adenocarcinomas of the rectum presenting without recurrent disease were additionally selected to match those presenting with recurrent disease. Individual control group patients matched patients in the local recurrence group, the distant recurrence group and the local & distant recurrence group. For every patient within a recurrence group (local, distant, or local & distant) a unique match was therefore included. Matching criteria were TNM stage (exact match), CRM involvement (exact match), gender (exact match), and age at surgery (average difference of 2 years; range 0-7 years).

For the discovery phase, we selected tissues from 112 rectal cancer patients for whom fresh-frozen (FF) tumor and normal matched tissues were available. These included samples from patients with a recurrence (all available local N = 10, distant N = 41, and all available local & distant N = 15) and matched control patients without a recurrence (N = 46). Three samples from the discovery cohort were used for technical validation. For validation of results, we selected tumor and normal matched formalin-fixed paraffin-embedded (FFPE) tissue from 95 patients. Patients presenting with a recurrence (all available local, N = 12, distant, N = 24, and all available local & distant, N = 22) were matched to controls (without any recurrence, N = 37). The discovery study cohort (N = 112) and validation cohort (N = 95), which did not overlap, are described in Table 1.

**Table 1:** **Summary of rectal cancer study cohorts**

	**Discovery cohort (FF)**		**Validation cohort (FFPE)**	
	**N = 112**	**Perc.**	**N = 95**	**Perc.**
**Age at surgery**				
<50	14	13%	4	4%
50-75	72	64%	72	76%
≥75	26	23%	19	20%
**Gender**				
Male	73	65%	57	60%
Female	39	35%	38	40%
**TNM stage**				
Stage I	11	10%	7	7%
Stage II	24	21%	21	22%
Stage III	77	69%	67	71%
**CRM involvement**				
No	82	73%	56	59%
Yes	30	27%	39	41%
**Recurrence**				
No recurrence	46	41%	37	39%
Local^a,b^	10	9%	12	13%
Distant^b^	41	37%	24	25%
Local & Distant^a,b^	15	13%	22	23%

Abbreviations: ^a^All available patients with fresh frozen/FFPE tumor and normal tissue specimens were included; ^b^Patients with a recurrence were matched to patients without a recurrence based on the matching criteria TNM stage, CRM involvement, gender, and age at surgery; N = Number of patients; Perc. = Percentage of total patients.

### Consent

All samples were coded in accordance with national ethical guidelines (“Code for Proper Secondary Use of Human Tissue”, Dutch Federation of Medical Scientific Societies). The use of these specimens was approved by the Medical Ethical Committee of the Leiden University Medical Center (LUMC). Patients were included in The Dutch Total Mesorectal Excision (TME) trial after informed consent was obtained [1].

### DNA isolation

Genomic DNA (gDNA) from tumor and corresponding normal tissue (FF or FFPE) was isolated, as described previously [14,15], following macrodissection or laser capture microdissection (LCM). The DNA concentration was measured using the PicoGreen method (Invitrogen, Carlsbad, CA, USA). If necessary, samples were concentrated using a Speedvac (SC110A, ThermoFisher, Waltham, MA, USA) to 10 ng/μl or to a minimum of 15 μl.

### CytoSNP arrays and analysis

Hybridization of gDNA to high resolution Illumina Human CytoSNP12v2 arrays (Illumina, San Diego, CA, USA), intensity data extraction and the first quality control steps were performed by ServiceXS (Leiden, the Netherlands). Bioinformatic analysis was performed using the beadarraySNP package in R [13]. After normalization, de-waving and automated segmentation analysis, we obtained CN data and allelic ratio data for all arrays. Thresholds for CN alterations after normalization were set at 0.92 for losses and 1.08 for gains, compared to the sample mean. To designate the allelic ratio groups, DNA segments were divided per individual sample into three classes, retention of heterozygosity, imbalance and loss of heterozygosity (LOH).

A globaltest (R package globaltest [16,17]) was performed on both the continuous CN data and the overall allelic ratio group data separately to determine overall statistical differences between analysis groups, comparing local recurrence group (L-group) *versus* control group (C-group), distant recurrence group (D-group) *versus* C-group and local & distant recurrence group (LD-group) *versus* C-group. A significant global test on the overall CN data or allelic ratio group data resulted in a second global test on individual chromosome arms. If significant, the smaller underlying segments underwent a further global test to identify regions with significant differences between the analysis groups. Multiple testing correction was performed using the Benjamin-Hochberg (BH) method [18].

For allelic ratio group analyses of chromosome combinations, the overall chromosome status was classified into balanced = 1, imbalanced = 2 and LOH = 3. Overall chromosome status was defined as most abundant allelic ratio group number on the chromosome. In the chromosome combination analyses, the highest number of overall chromosome status was used. Differences between groups were assessed with Fisher’s exact test for count data.

### Survival analysis

Survival analysis was performed using the Cox proportional hazards model (R-package survival). Survival data were available for 12 years of follow-up. The mean follow-up time of the discovery cohort and the validation cohort were 6.6 years (range 0.07-13.4 years) and 5.7 years (range 0.09-13.6 years), respectively. Overall survival (OS) was defined as the time from surgery until death by any cause. Disease specific survival (DSS) was defined as the time from surgery until death by rectal cancer. Local recurrence-free period (LRFP) was defined as the time from surgery until the discovery of a local recurrence. Distant recurrence-free period (DRFP) was defined as the time from surgery until the discovery of a distant recurrence. Multivariate models included the predetermined clinically important covariates TNM stage, age at surgery, gender and circumferential margin involvement, irrespective of statistical significance.

### Dynamic array and analysis

Based on the results from the discovery phase, reference SNP (rs) identification numbers on chromosome 7 and 13 were extracted from the CytoSNP12 array. A search was performed for validated ABI-Taqman SNP assays, based on rs-numbers, in the SNP browser program (Applied Biosystems, Foster City, CA, USA) and 48 SNPs were selected for validation, with 16 on chromosome 7p, 16 on chromosome 7q and 16 on chromosome 13 (Additional file 1 S1). Selection was based on the highest percentage of heterozygosity (at least 40%) as determined in the genotype call for normal tissues (N = 112) in the discovery phase.

Taqman assays targeting the 48 selected SNPs were tested using the 96.96 BioMark Dynamic Array for quantitative Real-Time PCR (Fluidigm Corporation, San Francisco, CA, USA). On the 96.96 BioMark dynamic platform, Taqman SNP targets were assayed in duplicate and samples were assayed in triplicate (technical validation) or duplicate (validation) using 14 cycles of Specific Target Amplification for each sample replicate prior to the qPCR on the array. Assays were performed by ServiceXS. Fluidigm Real-Time PCR analysis software was used to extract cycle threshold (Ct) values, while Fluidigm SNP Genotyping analysis software was used to extract endpoint measurements at cycle 40 and for genotype calling. The linear derivative was used to determine Ct threshold values per SNP assay. Samples with a Ct >30 and missing values were set at Ct = 30. The number of starting DNA molecules per reaction chamber was computed using the following formula: # DNA molecules = 2^(26-Ct). In the 96.96 BioMark dynamic assay, a Ct value of 26 corresponds to one DNA molecule in the reaction chamber. Total sample molecule amount of the VIC (A-allele) and FAM (B-allele) channels was used for sample normalization. The allelic ratio, based on calculated amounts of DNA molecules rather than intensity, can be calculated by dividing the values for the VIC (A-allele) and FAM (B-allele) channels.

### Validation of results

Based on quality control, samples with a mean log2 value lower than 4, and patients with non-matching tumor and normal SNP genotypes, were excluded from analyses. The Welch Two Sample T-test was used to assess whether average allelic ratio values along chromosome 7p, chromosome 7q or chromosome 13 were significantly different between the recurrence groups and the control group. Survival analysis for validation of results was performed as described above.

## Results and discussion

### Description of frequently occurring copy number alterations, and meta-analysis

**Figure 1:**
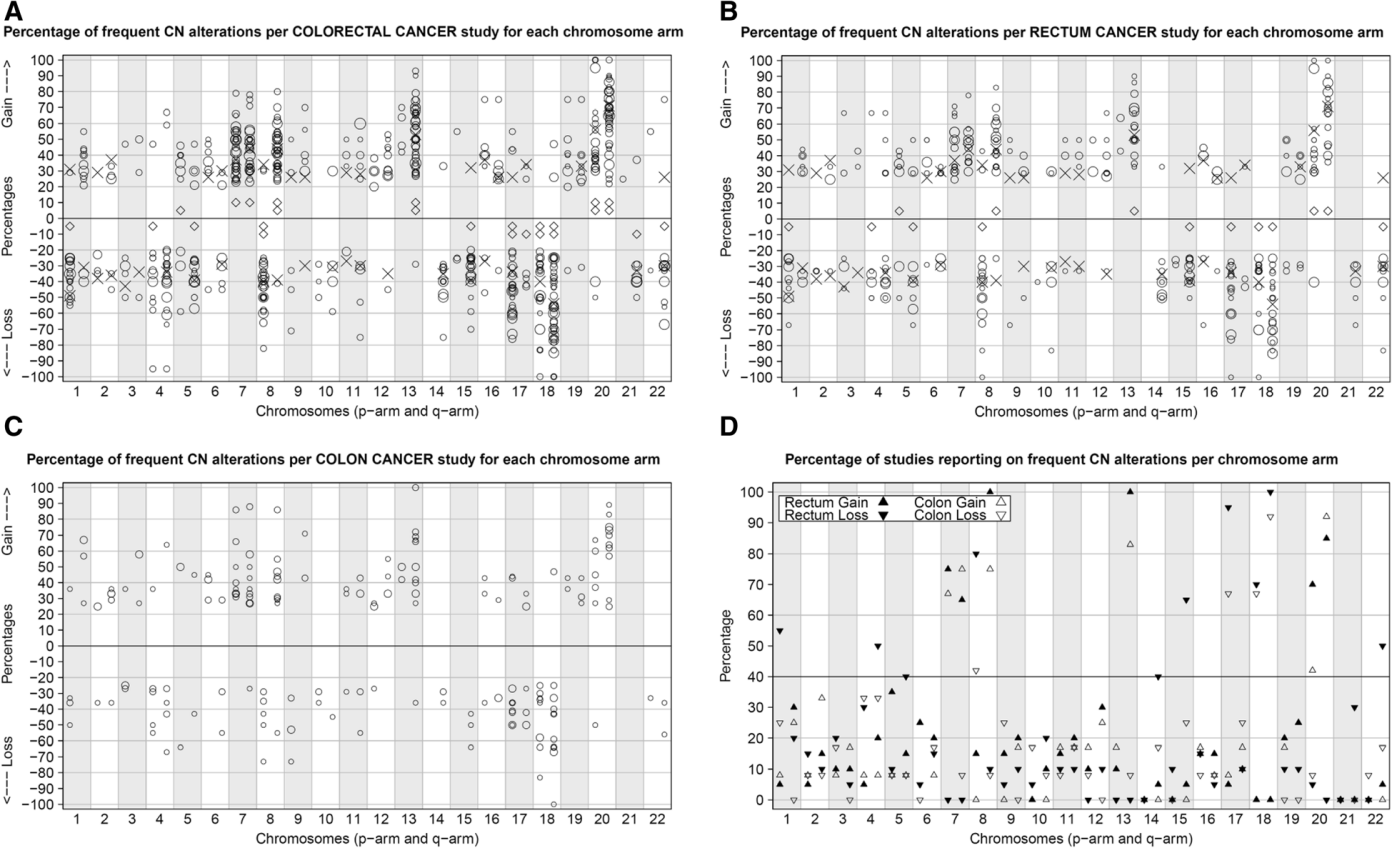
Frequent copy number alterations reported in colorectal cancer studies. Frequent CN alterations were defined as copy number (CN) changes found in at least 25% of the cases in a study cohort. Since most studies did not report low frequency CN alterations, only high frequency CN alterations were summarized. A-C. Percentage of high frequency CN alterations for each chromosome arm reported by different CGH/SNP studies for colorectal (**A**; plotted from 20% to 100%), rectum (**B**; plotted from 25% to 100%) or colon (**C**; plotted from 25% to 100%) cancer. Top panel: Percentages of gains. Lower panel; percentages of losses. Symbol size (circles/diamonds/X’s) indicates the number of rectal cases included in a particular study. X indicates the CN percentage from the current (SNP) study. Indicated losses are restricted to segments containing at least 15 SNPs. Open circles (O) represent other studies with reported percentages or percentages that could be estimated from plots or figures. For two studies [31,53], indicated by black diamonds (**◊**), percentages were not available. **D**. The percentage of studies, including the results of this study, were counted and plotted that reported on a chromosome arm showing CN alterations in ≥25% of the cases of that particular study. The percentages per chromosome arm for rectal studies are indicated with black-filled triangles (▲▼), while colon studies are indicated by open triangles (∆∇). Triangles that point upwards (▲∆) indicate the number of studies with reported gains and triangles that point downwards (▼∇) indicate the number of studies reporting on losses found on the various chromosome arms. We considered a particular CN alteration to be common, with high frequency, when at least 40% of the studies reported the CN alteration in at least 25% of the study’s cases.

We assessed CN alterations and chromosomal aberrations in rectal cancers from the Dutch TME trial, selected from the treatment arm that did not receive pRT. To gain a broader perspective on the results of our analysis of chromosomal CN alterations, we performed a meta-analysis of previously published CN studies. A PubMed search (performed on April 7, 2014), using the search criteria described in Additional file 2 S2, yielded 325 published studies. Studies from 2005 to search date and additional studies (selected from references of the selected articles published in 2005 or after) that described older CN alteration studies were assessed for eligibility, which required description of at least 6 tumors and detailed information on CN alterations per chromosome arm. A total of 42 studies that reported data on CN alterations in rectal cancer or colorectal cancer were included [11,19-59]. We combined results from these 42 studies (Table 2) and one additional study published online just after our Pubmed search (Table 2) and used the data to locate high frequency CN alterations in colorectal cancer at the level of chromosome arms (Figure 1A). CN alterations reported in at least 40% of the studies for at least 25% of the study cases were considered to be common CN alterations with high frequencies.

**Table 2:** **Colorectal cancer studies reporting on copy number alterations**

	**Studies**		**Origin of tissues**	**Method**	**Total CRC**		**Rectum**		**Colon**		**TNM stage**
					**Figure** **1** **A**	**#**	**Figure** **1** **B**	**#**	**Figure** **1** **C**	**#**	
1	Schlegel *et al.*	1995	Germany	CGH	yes	12	no	(x)	yes	12	
2	Ried *et al.*	1996	Germany	CGH	yes	16	yes	6	yes	10	2/3/(4)
3	Meijer *et al.*	1998	the Netherlands	CGH	yes	14	yes	7	yes	7	
4	Nakao *et al.*	1998	Japan	CGH	yes	9	no	0	yes	9	(1)/3
5	Paredes-Zaglul *et al.*	1998	USA	CGH	yes	9	yes	2	yes	7	(3)/4
6	Al-Mulla *et al.*	1999	UK	CGH	yes	12	no	(x)	no	(x)	2/3
7	De Angelis *et al.*	1999	Norway	CGH	yes	45	no	(x)	no	(x)	2/3/4
8	Georgiades *et al.*	1999	UK	CGH	yes	17	no	(11)	no	(6)	1/2/3
9	Korn *et al.*	1999	USA	CGH	yes	6	no	(0)	yes	6	2/3/4
10	Aust *et al.*	2000	USA/Germany	CGH	yes	42	no	(7)	no	(35)	1/2/3/4
11	Aragane *et al.*	2001	Japan	CGH	yes	30	no	(x)	no	(x)	1/2/3/4
12	Chan *et al.*	2001	China/UK	CGH	yes	16	no	(x)	no	(x)	2/3/4
13	De Angelis *et al.*	2001	Norway	CGH	yes	67	no	(x)	no	(x)	1/2/3/4
14	Nakao *et al.*	2001	Japan	CGH	yes	35	no	(13)	no	(22)	2/3/4
15	Rooney *et al.*	2001	USA	CGH	yes	29	no	(4)	no	(25)	3
16	Hermsen *et al.*	2002	the Netherlands	CGH	yes	82	no	(x)	no	(x)	1/(2)
17	Knösel *et al.*	2002	Germany	CGH	yes	15	no	(x)	no	(x)	
18	Alcock *et al.*	2003	UK	CGH	yes	17	yes	6	yes	11	
19	Ghadimi *et al.*	2003	Germany	CGH	yes	50	yes	18	yes	32	1/2/3
20	He *et al.*	2003	China	CGH	yes	26	yes	14	yes	12	2/3/4
21	Leslie *et al.*	2003	UK	CGH	yes	50	no	(21)	no	(29)	1/2/3
22	Bardi *et al.*	2004	Sweden/Denmark	CGH	yes	115	no	(51)	no	(63)	1/2/3/4
23	Diep *et al.*	2004	Norway/Sweden	CGH	yes	10	no	(x)	no	(x)	(3)/4
24	Nakao *et al.*	2004	Spain	aCGH	yes	125	no	(x)	no	(x)	1/2/3/4
25	Poeaim *et al.*	2005	Thailand	CGH	yes	40	no	(10)	no	(30)	
26	Tanami *et al.*	2005	Japan	CGH	yes	20	yes	9	yes	11	(2)/(3)/4
27	Al-Mulla *et al.*	2006	UK/Kuwait	CGH	yes	70	no	(47)	no	(18)	1/2
28	Grade *et al.*	2006	Germany	CGH	yes	21	yes	21	no	0	2/3
29	Grade *et al.* ^a^	2007	Germany	CGH	yes	32	no	0	yes	32	2/3
30	Lips *et al.*	2007	the Netherlands	SNPa	yes	77	yes	77	no	0	1/2/3
31	Xiao *et al.*	2007	China	CGH	yes	24	yes	9	yes	15	
32	Lips *et al.*	2008	the Netherlands	SNPa	yes	32	yes	32	no	0	1/2/(3)
33	Grade *et al.* ^a^	2009	Germany	CGH	yes	42	yes	42	no	0	1/2/3
34	Lagerstedt *et al.*	2010	Sweden	aCGH	yes	24	no	(x)	no	(x)	1/2/3/4
35	Molinari *et al.*	2011	Italy	aCGH	yes	51	yes	51	no	0	1/2/3
36	Nakao *et al.*	2011	Japan	aCGH	yes	94	no	(x)	no	(x)	1/2/3/4
37	Chen *et al.* ^b^	2011/2012	USA	aCGH	yes	95	yes	95	no	0	2/3
38	Kodeda *et al.*	2012	Sweden	aCGH	yes	16	yes	16	no	0	1/2/3
39	Shi *et al.*	2012	China	aCGH	yes	8	yes	8	no	0	
40	Liang *et al.*	2013	China	aCGH	yes	48	yes	48	no	0	2/3/4
41	Zhou *et al.*	2013	China	aCGH	yes	16	yes	16	no	0	2/3
42	Doyen *et al.*	2014	France/Germany	SNPa	yes	80	yes	80	no	0	(1/2/3)
43	This study	2014	the Netherlands	SNPa	yes	112	yes	112	no	0	1/2/3
	Total				43		20		12		

This table lists the published studies on CN alterations in rectal carcinomas used for comparisons with the present study (Nr. 43).

Abbreviations: CGH, comparative genome hybridization; aCGH, arrayCGH; SNPa, SNP array; ^a^Unclear if there is overlap with study Nr. 29 (Grade *et al.* [29]), ^b^Chen et al. published two papers based on the same patient cohort. Numbers in parentheses indicate the number of rectal or colon cancer patients included in the study, but for which results could not be distinguished between groups.

Many included studies pooled colon and rectal tumors and referred to them as colorectal cancer. However, evidence suggests that tumors arising from colon and rectal tissues should be considered as distinct disease entities [60-62]. We therefore compared common CN alterations with high frequencies in rectal versus colon cancers. Twenty studies (including the present study) specifically reported CN alterations in rectal carcinomas (Figure 1B). Frequent gains common in rectal cancer included regions of chromosomes 7p (found in 75% of the 20 rectal cancer studies, with a frequency >25%), 7q (65%), 8q (100%), 13q (100%), 20p (70%) and 20q (85%). Frequent losses common in rectal cancer were found on chromosomes 1p (found in 55% of the 20 rectal cancer studies, with a frequency >25%), 4q (50%), 5q (40%), 8p (80%), 14q (40%), 15q (65%), 17p (95%), 18p (70%), 18q (100%) and 22q (50%). Twelve studies reported CN alterations specific to colon carcinomas (Figure 1C), with frequent gains common in colon cancer on chromosomes 7p (found in 67% of the 12 studies, with a frequency >25%), 7q (75%), 8q (75%), 13q (83%), 20p (42%) and 20q (92%). Frequent losses common in colon cancer were found on chromosomes 8p (found in 42% of the 12 studies, with a frequency >25%), 17p (67%), 18p (67%) and 18q (92%). In contrast to rectal cancer, frequent losses on chromosomes 1p, 4q, 5q, 14q, 15q and 22q were not observed in at least 40% of the colon studies. With the exception of loss at 4q, these chromosome arms showed a difference of >20% between rectal cancer and colon cancer studies, indicating that rectal cancers were associated with a higher frequency of losses on these particular chromosome arms (Figure 1D). In addition, the percentage of studies reporting frequent losses on chromosomes 1q, 8p, 17p, and 21q, and the percentage of studies reporting frequent gains on chromosomes 8q and 20p, was higher in rectal cancer compared to colon cancer. These differences may be explained, in part, by the much higher percentage of microsatellite unstable colon carcinomas in comparison to rectal carcinomas, because microsatellite instability reflects DNA mismatch repair deficiency, in contrast to chromosomal instability characterized by aneuploidy. Where possible, microsatellite unstable tumors were omitted from our comparisons, although many studies did not report microsatellite stability status.

In conclusion, frequent CN alterations common in rectal cancer were located on chromosomes 1p, 4q, 5q, 7, 8, 13q, 14q, 15q, 17p, 18, 20 and 22q, while colon cancer showed a similar pattern but with a lower frequency of gains at chromosomes 8q and 20p, a lower frequency of losses at chromosomes 1q, 8p, 17p and 21q, and an absence of frequent CN losses at chromosomes 1p, 4q, 5q, 14q, 15q and 22q.

### Comparison of our study with the meta-analysis

In order to identify CN alterations associated with a recurrence in rectal cancer, we assessed CN alterations in a series of non-preoperatively treated rectal cancers from the Dutch TME trial. Rectal cancers with local and/or distant recurrences were compared with matching control cases without recurrence. In terms of the location of frequent gains, our study cohort was comparable to previously described rectal cancer cohorts (Figure 1B upper panel). In contrast, compared to previous studies we detected a higher number of chromosome arms with frequent losses (Figure 1B lower panel). This might be due to our use of high-density arrays rather than CGH arrays, and to the selection of previously studied CGH locations. Another explanation might be the larger sample size of our study compared to most of the earlier studies, as smaller studies are more prone to higher variance. Additionally, our study differs from other studies due to our use of both laser capture microdissection and macrodissection. This could have influenced results by reducing a potential effect in the analyses.

### Genomic abnormalities and local recurrence

Continuous CN values and allelic ratio groups were analyzed in an effort to identify differentially affected genomic regions in rectal cancer patients presenting with and without local and/or distant recurrence. We included all patients with a local recurrence for whom fresh frozen tissue was available (N = 25). Fifteen of these patients also presented with a distant recurrence. Additionally, we included 41 patients presenting with only distant recurrences. All patients with recurrent disease were matched to control samples of patients who did not present with a recurrence during follow-up (N = 46).

The frequency of gains (Additional file 3 S3A) and losses (Additional file 3 S3B) along the length of each chromosome was plotted for each analysis group (control group, local recurrence group, distant recurrence group, and local & distant recurrence group). CN gains were less frequently observed in the local recurrence group compared to the other analysis groups, while CN losses were more frequently observed in patients with a local recurrence. However, CN alterations were not significantly associated with (local) recurrent disease in our study cohort. A similar pattern can be deduced from a study by Diep *et al*. [49], with fewer CN gains and more CN losses in local recurrences compared to independent primary colorectal tumor samples. A lower frequency of gains along chromosome 7 and higher frequency of losses along chromosome 18q were especially associated with local recurrence in both studies. A study by Kodeda *et al.* [31], comparing locally recurrent and non-recurrent rectal tumors, reported that both CN gains and CN losses were less frequently observed in locally recurrent tumors. Lower percentages of CN gains on chromosomes 8q, 13q and 20q, and a lower frequency of CN losses on chromosome 18q were observed in locally recurrent tumors in both our study cohort and the Kodeda cohort. In addition, we observed a lower frequency of CN gains on chromosomes 9p, 16p, 17q, 19 and 22q in the locally recurrent tumors. In contrast to the Kodeda study, we observed a higher frequency of CN losses on chromosomes 1p and 5q in locally recurrent tumors, in addition to a higher frequency of losses on chromosomes 3p, 9p and 15q. Overall, the results of the Kodeda study were comparable with our study regarding the lower frequency of CN gains, but conflicting on frequency of CN losses. This difference might be a result of the higher number of small deletions identified in our study due to our use of high-density SNP arrays compared to the CGH arrays used by Kodeda *et al*. However, despite these differences both the Kodeda study [31] and our study share a common conclusion that none of the above described CN alterations are (statistically) significantly associated with local recurrent disease.

**Figure 2:**
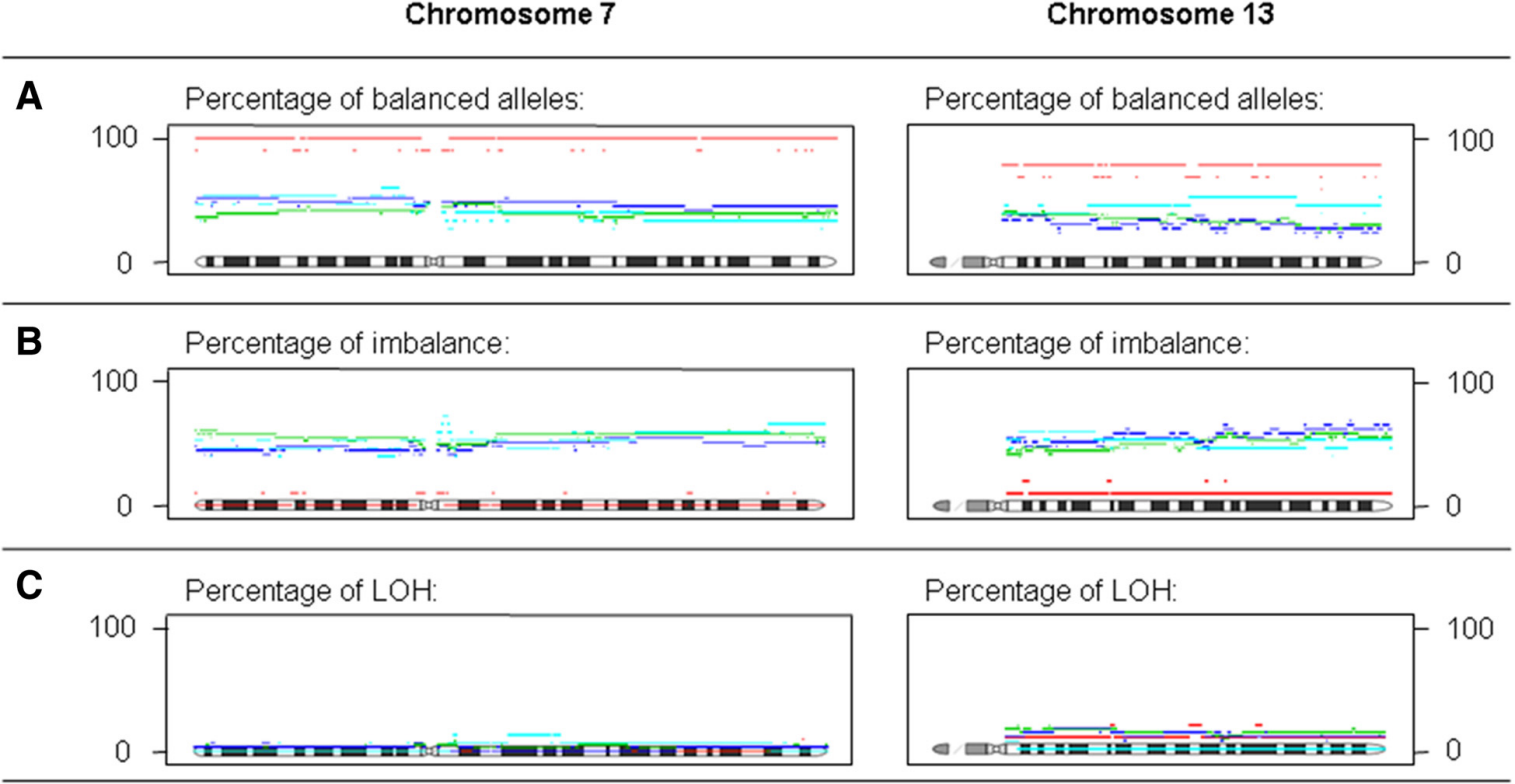
Distribution of the allelic ratio groups on chromosome 7 and 13 in the discovery phase. Legend: **- - - (Red broken line)** = local recurrence group, **- - - (Blue broken line)** = distant recurrence group, **- - - (Cyan broken line)** = local and distant recurrence group, **- - -** = control group. The percentage of patients, divided into four groups according their recurrence status, with tumors that contain balanced alleles **(A)**, imbalanced alleles **(B)**, and LOH **(C)**, spread along chromosomes 7 and 13.

An important feature of the SNP arrays used in our study was that they allow assessment of allelic imbalances, in addition to CN alterations. Using array data, allelic ratio groups were analyzed in order to identify genomic regions that showed allelic (im)balances associated with local and/or distant recurrence. The allelic ratio of DNA segments was classified into three classes: retention of heterozygosity, imbalance and LOH. In contrast to CN alterations, allelic imbalance showed a significant association with local recurrence, while no association was found with distant recurrence or a combination of both. Tumors from patients in the local recurrence group showed overall statistically significant differences in allelic ratios compared to the control samples (p < 0.005). Individual chromosome arms, and underlying segments and sub-segments, were then analyzed. Several chromosome arms showed different allelic ratio groups with (uncorrected) p-values <0.05 (Table 3A). In all cases, the local recurrence group displayed fewer allelic aberrations (allelic imbalance or LOH) than the control group. Statistically significant sub-regions, with p-values <0.05 after adjustment for multiple testing, were identified on chromosome 7. These regions showed almost no LOH (Additional file 4 S4). All tumors from patients presenting with only a local recurrence showed retention of heterozygosity and balanced alleles. In contrast, approximately 50 percent of control group cases showed imbalanced alleles. The percentage of patients with retention, imbalanced alleles or LOH along the length of chromosome 7 is shown in Figure 2. Balanced alleles were more prominent in cases with only a local recurrence, indicating that retention on chromosome 7 might be associated with the development of local recurrent disease. Frequent gains of chromosome 7, with or without allelic imbalances, were most often identified, and true LOH was almost non-existent in our study cohort. This suggests that heterozygosity of chromosome 7 is important for rectal cancer tumorigenesis and warrants further studies on the role of chromosome 7 retention in local recurrences of rectal cancer.

**Table 3:** **Differential allelic ratio groups between local recurrence and control**

**A)**			**Local recurrence group**			**Control group**		
**Chr.**	**p-value**	**BH**	**Retention**	**Imbalance**	**LOH**	**Retention**	**Imbalance**	**LOH**
1q	0.05	0.17	89%	11%	0%	45%	53%	2%
4q	0.05	0.17	78%	22%	0%	47%	22%	31%
**7p**	**0.0005**	**0.015**	**100%**	**0%**	**0%**	**36%**	**62%**	**2%**
**7q**	**0.0007**	**0.015**	**100%**	**0%**	**0%**	**9%**	**13%**	**78%**
10p	0.04	0.17	89%	11%	0%	53%	45%	2%
11p	0.04	0.17	89%	11%	0%	49%	42%	9%
13q	0.04	0.17	78%	11%	11%	27%	60%	13%
14q	0.017	0.17	78%	22%	0%	40%	22%	38%
15q	0.03	0.17	78%	22%	0%	29%	29%	42%
16q	0.06	0.18	89%	11%	0%	53%	36%	11%
17p	0.03	0.17	22%	33%	44%	9%	13%	78%
18q	0.05	0.17	11%	22%	67%	9%	13%	78%
22q	0.04	0.17	78%	11%	11%	33%	31%	36%
**B)**			**Local recurrence group**			**Control group**		
**Chr.**	**p-value**		**Retention**	**Imbalance**	**LOH**	**Retention**	**Imbalance**	**LOH**
7	0.0025		9	0	0	19	25	1
13	0.0062		7	1	1	12	27	6
7 + 13	<0.0002		7	1	1	5	33	7

A) For comparison of the local recurrence group and control group, chromosome arms are listed that showed significant allelic ratio groups with p-values <0.05 and adjusted p-values <0.2.

Abbreviations: Chr, chromosome; B, p-value after using the Benjamin-Hochberg method for multiple testing correction.

B) For chromosome 7, chromosome 13 and in combination, the numbers of patients within each ‘overall chromosome status’ group - defined as the most abundant allelic ratio group on the chromosome - are shown for both the local recurrence group (L) and the control group (C). Fisher’s exact test for count data was used to determine the statistical differences between analysis groups L and C.

The use of heterozygous SNPs to determine allelic ratios of normal and tumor samples identified statistically significant differences on chromosome 7 between patients presenting with and without only local recurrent disease.

### Validation

A validation of the association of chromosome 7 retention with local recurrence was performed using the 96.96 BioMark dynamic platform. A technical validation demonstrated that this platform could be used for validation of our results (Additional file 5 S5). We selected all (remaining) patients with a local recurrence for whom FFPE tissue was available (N = 34). Of these patients, 22 also presented with a distant recurrence. Additionally, we included 24 patients presenting with only distant recurrences. All patients with recurrent disease were matched to control patient samples without recurrence in follow-up (N = 37).

Differences in frequency of allelic (im)balances of specific SNPs on chromosome 7 were assessed in FFPE tissues of rectal cancer patients presenting with and without local and/or distant recurrence. Based on the results from the discovery phase, we selected 32 SNPs located on chromosome 7p (N = 16) and chromosome 7q (N = 16) (Additional file 1 S1). As a high percentage of patients presenting with only a local recurrence also showed retention on chromosome 13 (Figure 2), an additional set of 16 SNPs was selected on this chromosome to potentially increase discriminative power. SNPs on both chromosome 13 and chromosome 7 were therefore included to enhance differences between analysis groups. During the discovery phase, the overall chromosome status of combined chromosomes 7 and 13 was significantly different in patients in the local recurrence group compared to the control group (Fisher’s exact test for count data, p-value < 0.0002; Table 3B). Balanced alleles were more prominent in cases with a local recurrence, indicating that retention on chromosome 7, and to a lesser extent on chromosome 13, might be associated with the development of a local recurrence.

**Figure 3:**
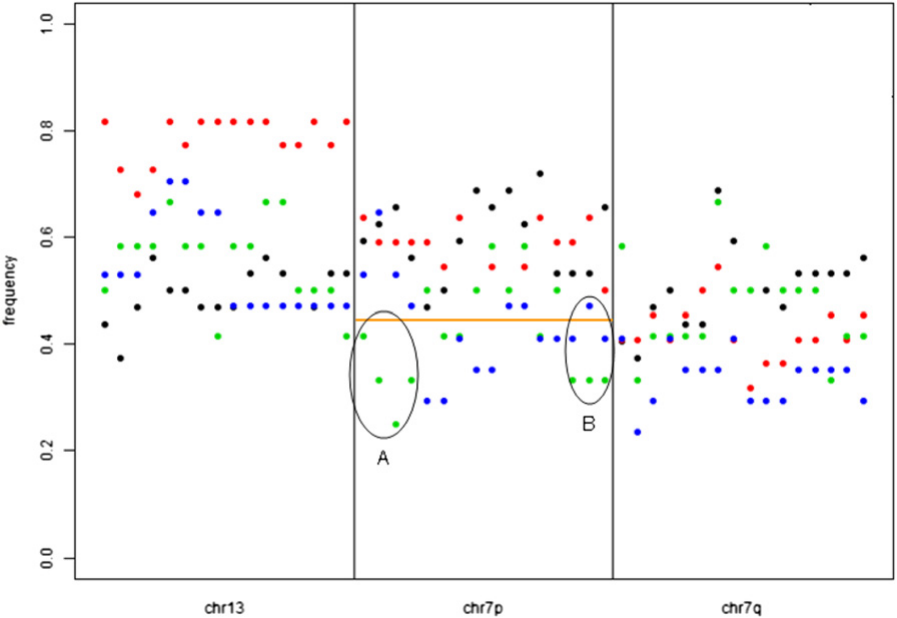
Frequency of allelic imbalance or LOH at validated loci. The percentage of patients with loss (imbalanced alleles or LOH), spread along chromosomes 7 and 13. All SNPs below the orange line (**— Orange Line**) represent SNPs that presented with lower percentages of patients in only the L or LD groups. X-axis = from left to right, locations of SNPs along the chromosome (arms) Y-axis = the percentage of patients with imbalanced alleles or LOH (0 = > 0%, 1 = > 100%) ● = C group, (Red circle) = D group, (Green circle) = L group, (Blue circle) = LD group. **A)** Location on chromosome 7p (telomeric region) where the L group in particular showed a lower percentage of patients with imbalanced alleles or LOH compared to the other groups. **B)** Location on chromosome 7p (centromeric region) where both the L group as well as the LD group showed a lower percentage of patients with imbalanced alleles or LOH compared to the other groups.

Using the technically validated dynamic array approach, differences between the local recurrence group and the control group showed a trend towards significance on chromosome 7p (p-value = 0.07). Retention of heterozygosity was more frequently observed in tumors with local recurrence compared to control group tumors, which is in accordance with findings in the discovery phase (Figure 3). The telomeric region of chromosome 7p showed allelic imbalance or LOH in consistently lower percentages of patients in the local recurrence group compared to the control group. For the centromeric region, the same pattern was observed for both the local recurrence group and for the local & distant recurrence group. Chromosome 13 retention results could not be reproduced.

In brief, validation cohort data were not conclusive but do support the notion that retention of heterozygosity on the telomeric and centromeric regions of chromosome 7 may be associated with local recurrence in rectal cancer.

### Prognostic value of CN alterations and allelic aberrations

The clinical prognostic value of continuous CN profiles and allelic ratio group profiles was assessed in the discovery cohort using the Cox proportional hazards model. Multivariate analyses showed a trend in association of chromosome 7p with local recurrence (LRFP), but no associations with any other clinical outcome in our cohort of rectal cancer patients (Additional file 6 S6). In our discovery study cohort, allelic imbalance was associated with death by rectal cancer and local recurrent disease, with the allelic imbalance at chromosome 7p being chiefly responsible for the prognostic effect for local recurrent disease (Additional file 6 S6). These results could not be confirmed with the 48 selected SNPs using the dynamic array (data not shown), and this outcome is reflected in the absence of literature describing associations between CN alterations or allelic ratio profiles and patient survival in rectal cancer patients.

Only one study specifically dedicated to rectal cancer patients reported an association of CN alterations with patient survival or recurrent disease [63]. The study by Doyen *et al.* [63] showed (in multivariate analysis) an association of loss at chromosome 8p with worse cancer specific survival (CSS) and the occurrence of metachronous distant metastases (DRFP). Unfortunately, the covariates included in the multivariate analysis where not reported. Four previously published colorectal cancer studies reported associations of CN alterations with patient survival or recurrent disease [11,45,48,64]. A study by De Angelis *et al*. [48] showed worse overall survival for patients with losses at chromosomes 1p and 8p in multivariate analyses. Bardi *et al*. [45] observed a significant association between loss at chromosome 4 and worse disease-free survival in univariate analyses. In multivariate analysis, loss of chromosome 18 was reported to be associated with worse overall survival. Al-Mulla *et al*. [11] reported that loss at chromosomes 4p and 5q was associated with worse disease-free survival in early stage colorectal cancers in multivariate analyses. Allelic imbalance at chromosome 8p was reported by Halling *et al.* [64] and associated with overall survival (OS) and time to recurrence (DRFP) in multivariate analysis. Association between allelic imbalance or loss at chromosome 8p and worse clinical outcome was described in three independent articles. However, only one was dedicated specifically to rectal cancer, and our study could not validate the results. The differences in results between these studies and our own data might be due to differences in tumor locations, as we focused on rectal cancer patients alone, whereas four out of five earlier studies included both colon and rectal cancer patients. Additionally, our use of laser capture microdissection along with macrodissection in order to reduce the potential effect of tumor microenvironment on the analyses, and thereby reduction of intratumoral heterogeneity of the rectal tumors, might provide a truer picture of the association between CN alterations and clinical outcome.

Allelic aberrations and LOH in colorectal cancer have been widely investigated, and loss of 18q and 17p are seen as prognostic markers for clinical outcomes in colorectal cancer (reviewed in [65]). To the best of our knowledge, no study has previously focused specifically on rectal cancers. A study by Choi et al. [66] showed that higher levels of LOH were significantly associated with tumor location specifically in the rec and the distal portion of the colon. This finding indicates that rectal and colon tumors should be considered distinct disease entities in relation to allelic alterations and LOH in particular. Prognostic indicators identified in studies of colorectal cancer patients cannot be adequately compared with (our) survival data on rectal cancer patients.

## Conclusion

### Implications for clinical use

Rectal cancer is associated with a high rate of local recurrence, in contrast to colon cancer. The location of the rectum, fixed in the smaller pelvis, provides opportunities for pRT or pCCRT treatment. Although introduction of pRT and pCCRT led to markedly reduced local recurrence rates (from 11% to 5% for pRT [2]), no difference in overall survival was observed [2,4,8]. This suggests that the majority of rectal cancer patients (over 90%) are currently receiving unnecessary preoperative treatment to reduce the local recurrence risk, when in fact this risk is only relevant for less than 10% of all patients. Identification of patients who are likely to show local recurrent disease could guide decision-making for the preoperative treatment of rectal cancer patients.

Validation data from the present study on allelic ratios of chromosome 7 are supportive (but not conclusive) of our initial finding that retention of heterozygosity on chromosome 7 is associated with local recurrent disease. While these data do not yet provide sufficient grounds for development of a clinically useful platform based on these observations, further research is warranted to elucidate underlying mechanisms. Chromosome 7 harbors many interesting genes, including druggable targets such as the oncogenes *EGFR, BRAF* and *MET*, but at present their relation to the retention of chromosome 7 and the development of local recurrent disease in rectal cancer is unclear. Comparison of data on CN alterations and allelic imbalance from our rectal cancer cohort with previously published studies provided support for the hypothesis that colon cancer and rectal cancer may be distinct disease entities, and thus may require stratification in (survival) analyses accordingly.

## Additional files

Additional file 1:
**Validation SNPs.**
Additional file 2:
**Search criteria for meta-analysis.**
Additional file 3:
**Frequency plots for CN gains.**
Additional file 4:
**Statistically significant regions in allelic ratio on chromosome 7q.**
Additional file 5:
**Technical validation of SNPs.**
Additional file 6:
**Overall clinical prognostic value for copy number and allelic ratio.**
