# Supplementary material for: Copy number alterations and allelic ratio in relation to recurrence of rectal cancer
Source: BMC Genomics. 2015 Jun 6;16(1):438. doi: 10.1186/s12864-015-1550-0 (PMC4458034; doi:10.1186/s12864-015-1550-0)
Supplement: Additional file 1: — Validation SNPs. [file 12864_2015_1550_MOESM1_ESM.doc]

**Supplementary File S1.** Validation SNPs


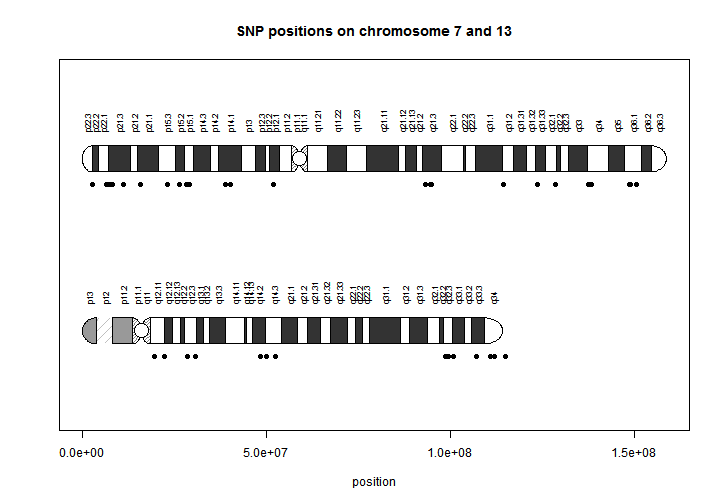


Shown are the locations of the SNPs selected for validation on chromosomes 7 and 13.

Validation SNP locations and frequencies of heterozygosity in the normal samples

| SNP | chr | bp position | discovery  cohort % | validation  cohort % |
| --- | --- | --- | --- | --- |
| rs9511368 | 13 | 19635339 | 52.3 | 45.5 |
| rs2274296 | 13 | 22255301 | 53.6 | 54.0 |
| rs3897927 | 13 | 28562901 | 54.5 | 55.2 |
| rs663528 | 13 | 30607076 | 54.5 | 54.6 |
| rs9534862 | 13 | 48480516 | 51.8 | 56.8 |
| rs6561522 | 13 | 49944530 | 49.1 | 47.1 |
| rs2247119 | 13 | 50087142 | 53.6 | 50.0 |
| rs2031992 | 13 | 52506372 | 52.7 | 51.1 |
| rs555647 | 13 | 98632349 | 54.5 | 44.8 |
| rs772311 | 13 | 99468479 | 55.4 | 51.1 |
| rs7318781 | 13 | 100878820 | 49.1 | 55.8 |
| rs1407584 | 13 | 107139790 | 45.5 | 36.1 |
| rs2298239 | 13 | 110815400 | 51.8 | 56.8 |
| rs7998332 | 13 | 111800441 | 53.6 | 48.9 |
| rs3783083 | 13 | 111956103 | 55.4 | 46.0 |
| rs4063 | 13 | 114861102 | 54.5 | 37.9 |
| rs1036504 | 7p | 2609765 | 53.6 | 35.6 |
| rs836508 | 7p | 6456669 | 50.9 | 42.1 |
| rs1806552 | 7p | 6656897 | 52.7 | 41.4 |
| rs1638214 | 7p | 7316977 | 53.6 | 54.0 |
| rs10243846 | 7p | 8062590 | 54.5 | 53.4 |
| rs3779363 | 7p | 8253685 | 54.5 | 37.9 |
| rs6954805 | 7p | 11144030 | 46.4 | 25.0 |
| rs6461202 | 7p | 15713934 | 46.4 | 50.0 |
| rs1558313 | 7p | 23156079 | 52.7 | 47.7 |
| rs2699810 | 7p | 26403574 | 52.7 | 39.8 |
| rs177465 | 7p | 28302072 | 55.4 | 46.6 |
| rs13246846 | 7p | 29160852 | 55.4 | 58.0 |
| rs4610638 | 7p | 38809937 | 48.2 | 46.6 |
| rs6951375 | 7p | 40248683 | 53.6 | 50.0 |
| rs997556 | 7p | 51964745 | 51.8 | 50.0 |
| rs1993956 | 7p | 51970823 | 52.7 | 42.1 |
| rs2074120 | 7q | 93107093 | 56.3 | 46.0 |
| rs6970823 | 7q | 93300636 | 56.3 | 59.1 |
| rs1326147 | 7q | 94471104 | 48.2 | 56.8 |
| rs854738 | 7q | 94615259 | 49.1 | 46.6 |
| rs854726 | 7q | 94626992 | 52.7 | 48.8 |
| rs854539 | 7q | 94915975 | 55.4 | 42.1 |
| rs12705973 | 7q | 114313199 | 54.5 | 44.8 |
| rs10255200 | 7q | 123679743 | 47.3 | 38.6 |
| rs339057 | 7q | 128376900 | 56.3 | 50.0 |
| rs339054 | 7q | 128382511 | 51.8 | 53.4 |
| rs889869 | 7q | 137506041 | 53.6 | 50.0 |
| rs11770967 | 7q | 138346011 | 54.5 | 51.1 |
| rs10252308 | 7q | 148501703 | 54.5 | 52.3 |
| rs1105261 | 7q | 148794442 | 52.7 | 47.7 |
| rs11525060 | 7q | 148801091 | 48.2 | 42.1 |
| rs3173833 | 7q | 150491084 | 53.6 | 54.0 |

Listed are the locations and frequencies of the SNPs selected for validation of results on chromosomes 7 and 13.

Abbreviations: chr= chromosome, bp= base pair
