# Supplementary material for: Copy number alterations and allelic ratio in relation to recurrence of rectal cancer
Source: BMC Genomics. 2015 Jun 6;16(1):438. doi: 10.1186/s12864-015-1550-0 (PMC4458034; doi:10.1186/s12864-015-1550-0)
Supplement: Additional file 2: — Search criteria for meta-analysis. [file 12864_2015_1550_MOESM2_ESM.doc]

**Supplementary File S2.** Search criteria for meta-analysis

("rectal neoplasms"[MeSH Terms] OR ("rectal"[All Fields] AND "neoplasms"[All Fields]) OR "rectal neoplasms"[All Fields] OR ("rectum"[All Fields] AND "cancer"[All Fields]) OR "rectum cancer"[All Fields] OR "rectal carcinoma"[All Fields] OR ("rectum"[All Fields] AND "carcinoma"[All Fields])) AND (("chromosome aberrations"[MeSH Terms] OR ("chromosome"[All Fields] AND "aberrations"[All Fields]) OR "chromosome aberrations"[All Fields] OR ("chromosomal"[All Fields] AND "aberrations"[All Fields]) OR "chromosomal aberrations"[All Fields]) OR ("dna copy number variations"[MeSH Terms] OR ("DNA"[All Fields] AND "copy"[All Fields] AND "number"[All Fields] AND "variations"[All Fields]) OR "dna copy number variations"[All Fields] OR ("copy"[All Fields] AND "number"[All Fields] AND "variation"[All Fields]) OR "copy number variation"[All Fields]) OR ("copy" [All Fields] AND "number" [All Fields] AND "changes"[All Fields]) OR ("SNP array"[All Fields] OR "SNP-array"[All Fields]) OR "CGH"[All Fields] OR "comparative genomic hybridization"[All Fields])

Results: 325 publications on 7^th^ of April 2014
