# Supplementary material for: Copy number alterations and allelic ratio in relation to recurrence of rectal cancer
Source: BMC Genomics. 2015 Jun 6;16(1):438. doi: 10.1186/s12864-015-1550-0 (PMC4458034; doi:10.1186/s12864-015-1550-0)
Supplement: Additional file 3: — Frequency plots for CN gains. [file 12864_2015_1550_MOESM3_ESM.doc]

**Supplementary File S3A.** Frequency plots for CN gains

**
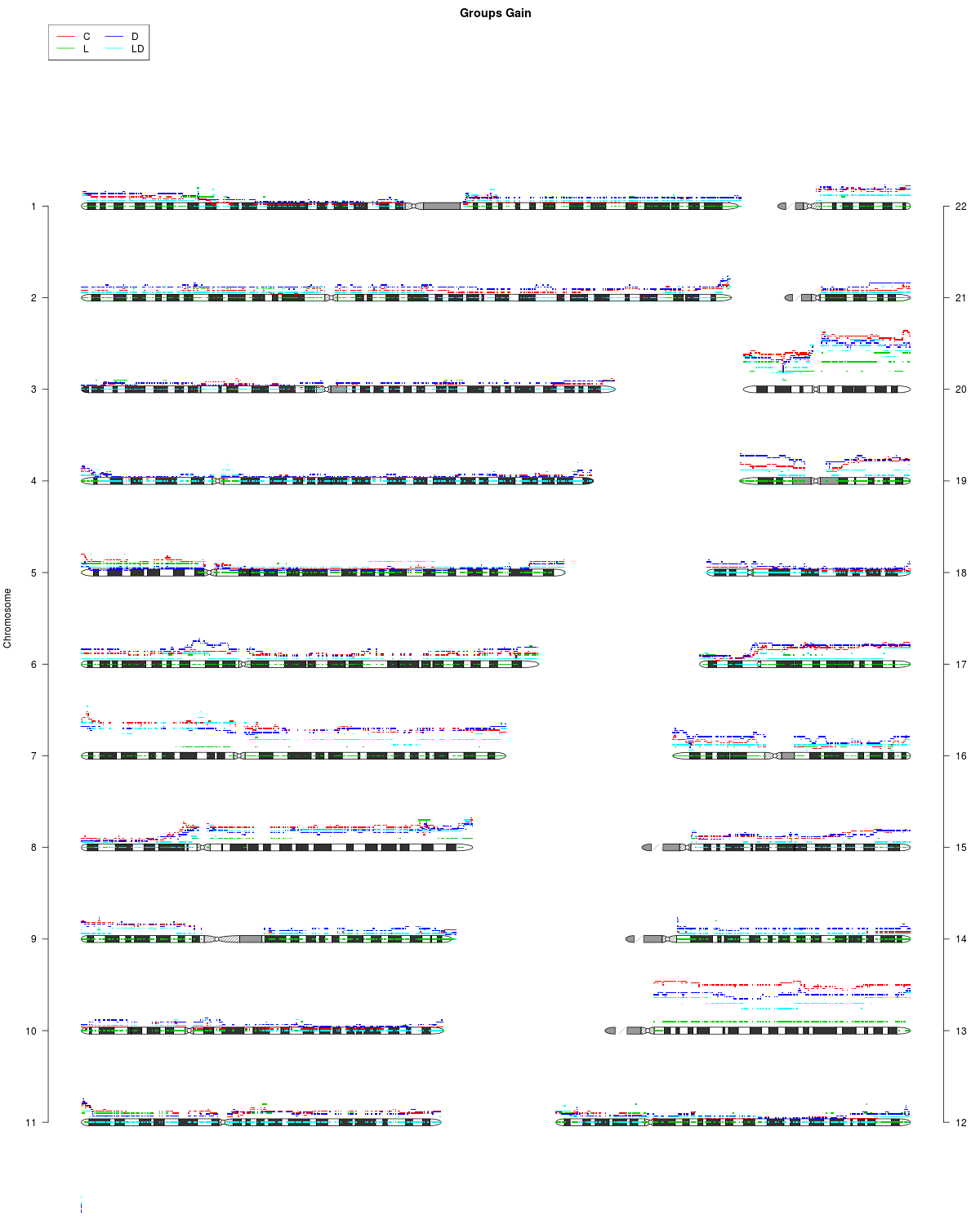
**

This plot shows the frequency of gains along the length of the chromosomes.

Abbreviations: C= control group, D= distant recurrence group, L= local recurrence group, LD= group with local & distant recurrences.

**Supplementary File S3B.** Frequency plots for CN losses

**
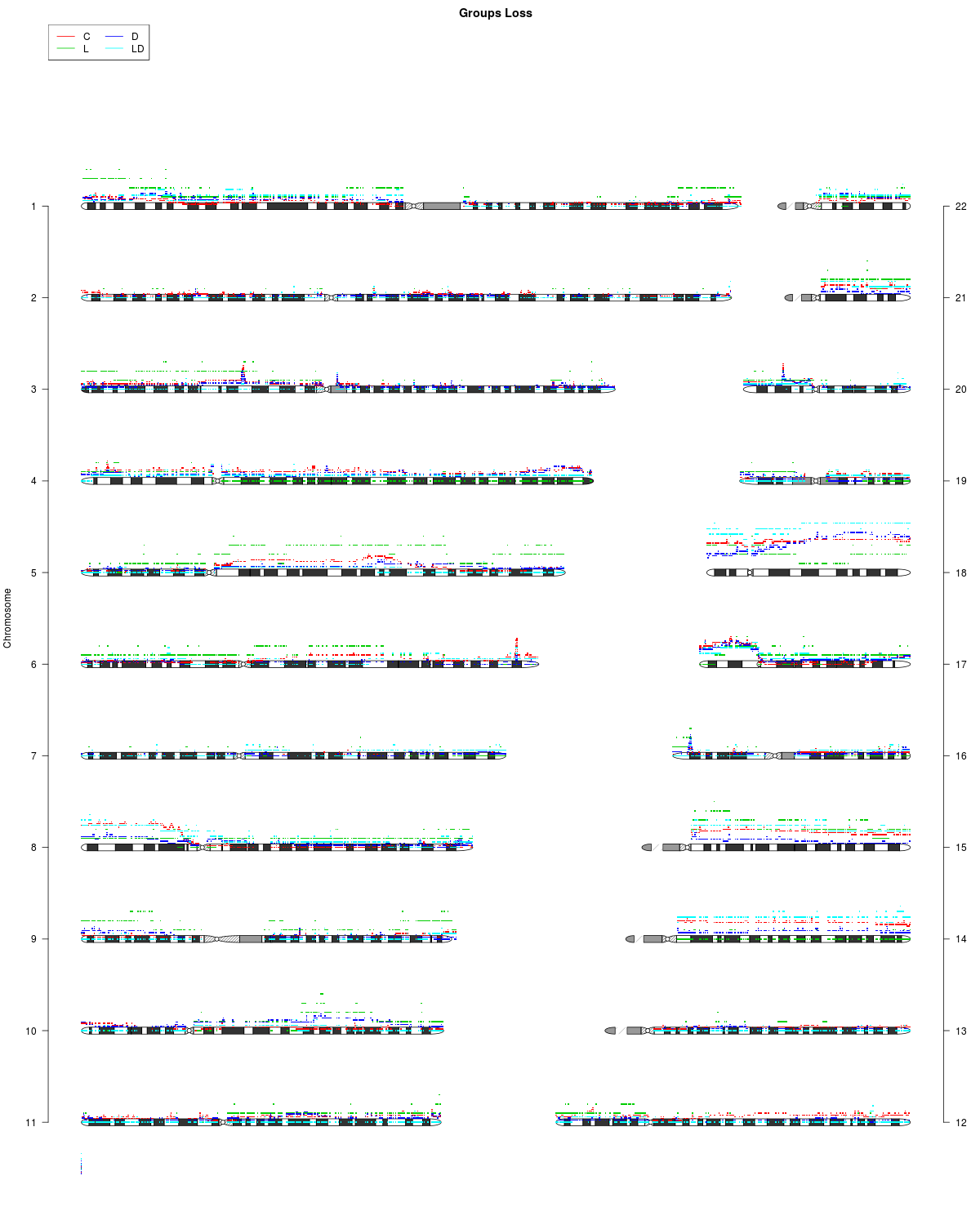
**

This plot shows the frequency of losses along the length of the chromosomes.

Abbreviations: C= control group, D= distant recurrence group, L= local recurrence group, LD= group with local & distant recurrences.
