# Supplementary material for: Copy number alterations and allelic ratio in relation to recurrence of rectal cancer
Source: BMC Genomics. 2015 Jun 6;16(1):438. doi: 10.1186/s12864-015-1550-0 (PMC4458034; doi:10.1186/s12864-015-1550-0)
Supplement: Additional file 4: — Statistically significant regions in allelic ratio on chromosome 7q. [file 12864_2015_1550_MOESM4_ESM.doc]

**Supplementary File S4.** Statistically significant regions in allelic ratio on chromosome 7q

| arm | p-value | BH | start position | end position |
| --- | --- | --- | --- | --- |
| 7q | 0.0003582676 | 0.014689 | 75632496 | 75691659 |
| 7q | 0.0003582676 | 0.014689 | 75702144 | 75768725 |
| 7q | 0.0003582676 | 0.014689 | 75779876 | 75910682 |
| 7q | 0.0003582676 | 0.014689 | 75955687 | 77623527 |
| 7q | 0.0003582676 | 0.014689 | 77624210 | 77734491 |
| 7q | 0.0003582676 | 0.014689 | 77754974 | 89349985 |
| 7q | 0.0003582676 | 0.014689 | 89372542 | 99083850 |
| 7q | 0.0003582676 | 0.014689 | 99446493 | 100386197 |
| 7q | 0.0003582676 | 0.014689 | 100391501 | 100397867 |
| 7q | 0.0003582676 | 0.014689 | 100401971 | 100770428 |
| 7q | 0.0003582676 | 0.014689 | 100774904 | 101006914 |
| 7q | 0.0003582676 | 0.014689 | 101012950 | 101073344 |
| 7q | 0.0003582676 | 0.014689 | 101093260 | 104070756 |
| 7q | 0.0003582676 | 0.014689 | 104077094 | 104259883 |
| 7q | 0.0003582676 | 0.014689 | 104267893 | 104367167 |
| 7q | 0.0003582676 | 0.014689 | 104371646 | 105201158 |
| 7q | 0.0003582676 | 0.014689 | 105219112 | 106078354 |
| 7q | 0.0003582676 | 0.014689 | 106102947 | 109272233 |
| 7q | 0.0003582676 | 0.014689 | 109355583 | 111075216 |
| 7q | 0.0003582676 | 0.014689 | 111104312 | 116958010 |
| 7q | 0.0003582676 | 0.014689 | 116962379 | 134043962 |
| 7q | 0.0003582676 | 0.014689 | 134046001 | 134098170 |
| 7q | 0.0003582676 | 0.014689 | 134108078 | 143782987 |
| 7q | 0.0003582676 | 0.014689 | 143795258 | 144310242 |
| 7q | 0.0003582676 | 0.014689 | 144452993 | 148761694 |
| 7q | 0.0003582676 | 0.014689 | 148929485 | 148992215 |
| 7q | 0.0003582676 | 0.014689 | 148994296 | 151177796 |
| 7q | 0.0003582676 | 0.014689 | 152962983 | 156401228 |
| 7q | 0.0003582676 | 0.014689 | 156404422 | 156609960 |
| 7q | 0.0003582676 | 0.014689 | 156956293 | 157683702 |
| 7q | 0.0003582676 | 0.014689 | 157690434 | 158812247 |
| 7q | 0.0003582676 | 0.014689 | 151422091 | 152781122 |

This table gives the locations on chromosome 7q that show statistically significant difference in allelic ratio between the local recurrence group and the control group.

Abbreviations: BH= p-value with multiple testing correction using the Benjamin-Hochberg method.
