# Supplementary material for: Copy number alterations and allelic ratio in relation to recurrence of rectal cancer
Source: BMC Genomics. 2015 Jun 6;16(1):438. doi: 10.1186/s12864-015-1550-0 (PMC4458034; doi:10.1186/s12864-015-1550-0)
Supplement: Additional file 5: — Technical validation of SNPs. [file 12864_2015_1550_MOESM5_ESM.doc]

**Supplementary File S5.** Technical validation of SNPs

A technical validation of the 96.96 BioMark dynamic platform was performed to explore the use of the dynamic array as a platform for validation of results. FF and FFPE tissues of 3 patients previously assayed in the discovery phase were used for the technical validation of allelic ratio differences on chromosome 7 and 13. Samples were selected based on their phenotype call in the discovery phase. In this collection of samples all allelic ratio groups were represented, being retention of heterozygosity or imbalanced alleles or LOH, for both chromosome 7 and 13.

Based on the results from the discovery phase we selected 48 SNPs, located on chromosome 7p (N=16), chromosome 7q (N=16) and chromosome 13 (N=16) (Supplementary File S1). Using a dynamic array we assessed these 48 SNPs for agreement of allelic ratio group (retention, imbalance or LOH) per SNP between the SNP-arrays and the dynamic array. Allelic ratio was determined and plotted for assessment of performance compared to the discovery phase (Supplementary File S5A). Technical validation using the dynamic array showed concordance with the SNP-array results obtained for the same samples in the discovery phase (Supplementary File S5B), thereby confirming that the dynamic array may be used for validation of results.

A) Polar transform plots for SNPs on chromosome 7 or 13 in the technical validation phase

B) Comparison between SNP-array data and dynamic array data at the level of allelic ratio groups


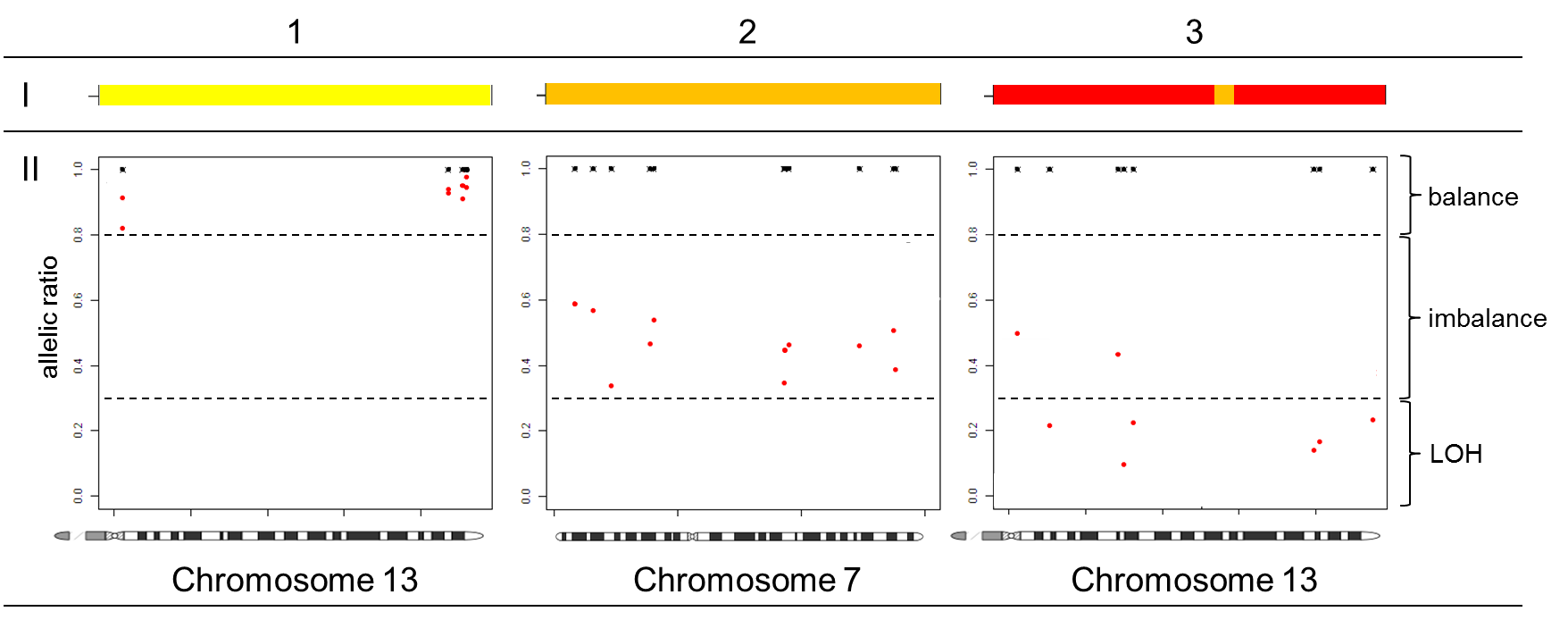


A) These polar transformed plots show how SNP intensity data of the dynamic array may be used to determine allelic ratio, and thereby allelic (im)balance or LOH. The y-axis represents the normalized molecule count of both the A- and B-allele channels. The x-axis represents the B-allele ratio (FAM/total value). Data points are colored if the normal sample showed heterozygous SNP expression. At x ≈ 0 and x ≈ 1 samples are homozygous for one of the alleles. At x ≈ 0.5, samples have balanced alleles.

Example are shown of retention of chromosome 7 (rs177465; technical validation sample #1), imbalance on chromosome 13 (rs2247119; sample #2) and LOH on chromosome 13 (rs2031992; sample #3).

B) This plot compares allelic ratio group data of the discovery phase (top panel I) with the technical validation using the dynamic array (bottom panel II). In the top panel (I) balanced alleles are indicated with a yellow (■), imbalanced alleles are indicated in orange (■), and LOH is indicated in red (■). In the bottom panel (II) for each SNP, the percentage of deviation of the tumor sample (●) from the heterozygous normal tissue sample (●) was plotted. The range to be considered balanced, imbalanced or LOH are indicated (- - -). Plots 1 represent chromosome 13 of sample #1, plot 2 represents chromosome 7 of sample #3 and plot 3 represents chromosome 13 of sample #3.
