# Supplementary material for: Copy number alterations and allelic ratio in relation to recurrence of rectal cancer
Source: BMC Genomics. 2015 Jun 6;16(1):438. doi: 10.1186/s12864-015-1550-0 (PMC4458034; doi:10.1186/s12864-015-1550-0)
Supplement: Additional file 6: — Overall clinical prognostic value for copy number and allelic ratio. [file 12864_2015_1550_MOESM6_ESM.doc]

**Supplementary File S6.** Overall clinical prognostic value for copy number and allelic ratio

| **A**) | | Survival analyses | Copy number (continuous)  Adjusted p-value | | Allelic ratio (groups)  Adjusted p-value | |  |
| --- | --- | --- | --- | --- | --- | --- | --- |
|  | |  | univariate | multivariate | univariate | multivariate |  |
|  | Discovery | OS | 0.85 | 0.88 | *0.083* | *0.086* |  |
|  |  | DSS | 0.85 | 0.86 | *0.091* | **0.046** |  |
|  |  | LRFP | 0.15 | *0.084* | **0.032** | **0.012** |  |
|  |  | DRFP | 0.86 | 0.84 | 0.74 | 0.66 |  |
| **B**) |  | LRFP | Copy number (continuous)  multivariate | | Allelic ratio (groups)  multivariate | |  |
|  |  | Chr. | p-value | Adjusted  p-value | p-value | Adjusted  p-value | position |
|  | Discovery | 7q | **0.011** | 0.43 | na | na | whole arm |
|  |  | 7p | na | na | **0.0023** | *0.096* | 10264689–10353158 |
|  |  | 7p | na | na | **0.0023** | *0.096* | 10356737–10686855 |
|  |  | 7p | na | na | **0.0023** | *0.096* | 10761590–15487883 |
|  |  | 7p | na | na | **0.0023** | *0.096* | 18901835–19095649 |

This table shows the prognostic value of CN alterations and allelic ratio groups in global analyses (A; for OS, DSS, LRFP and DRFP) and for specific significant regions at individual chromosome arms (B; for LRFP) in the Discovery set. Significant results are indicated in bold, trends are indicated in italic.

Abbreviations: OS= overall survival, DSS= disease specific survival, LRFP= local recurrence-free period, and DRFP= distant recurrence-free period.
